# Effects of Compression Garments on Muscle Strength and Power Recovery Post-Exercise: A Systematic Review and Meta-Analysis

**DOI:** 10.3390/life15030438

**Published:** 2025-03-11

**Authors:** Xiang Li, Hao Su, Liwen Du, Gen Li, Yuanyuan Lv, Xiaojie Liu, Lin Feng, Laikang Yu

**Affiliations:** 1 Beijing Key Laboratory of Sports Performance and Skill Assessment, Beijing Sport University, Beijing 100084, China; jinaxun0889@yeah.net (X.L.); sunflowerlyy@bsu.edu.cn (Y.L.); 2 Department of Strength and Conditioning Assessment and Monitoring, Beijing Sport University, Beijing 100084, China; 15662755237@163.com (H.S.); duliwen2019@126.com (L.D.); 3 School of Physical Education & Sports Science, South China Normal University, Guangzhou 510631, China; li287270242@163.com; 4 China Institute of Sport and Health Science, Beijing Sport University, Beijing 100084, China; 5 Department of Pharmacology and Toxicology, Medical College of Wisconsin, Milwaukee, WI 53226, USA; xiaojieliu@mcw.edu; 6 School of Sport Sciences, Beijing Sport University, Beijing 100084, China; 7 Beijing Sports Nutrition Engineering Research Center, Beijing 100084, China

**Keywords:** compression garment, muscle strength, power, exercise-induced muscle fatigue

## Abstract

This study investigated the effects of compression garments on mitigating the decline in muscle strength and power resulting from exercise-induced muscle fatigue. Searches were performed in PubMed, Web of Science, EBSCO, Cochrane, and Scopus databases. The three-level restricted maximum likelihood random effects model was used to synthesize the data. Twenty-seven studies met the inclusion criteria. Compression garments had significant restorative effects on muscle strength (Hedges’s g = −0.21, *p* < 0.01) and power (Hedges’s g = −0.23, *p* < 0.01) after exercise-induced muscle fatigue. Subgroup analysis revealed that compression garments were effective in mitigating the decline in muscle strength when the rest intervals were 1–48 h and over 72 h and in mitigating the decline in power when the resting interval was 1–24 h. In addition, compression garments significantly mitigated the decline in muscle strength, during rest intervals of 1–24 h for trained individuals and over 72 h for both trained and untrained individuals, after exercise-induced muscle fatigue. In conclusion, compression garments significantly mitigated the decline in muscle strength after exercise-induced muscle fatigue. Both trained and untrained individuals could benefit from compression garments, with the effectiveness of compression garments being more pronounced in trained individuals compared to untrained ones.

## 1. Introduction

Exercise-induced muscle fatigue refers to a temporary, reversible decrease in muscle contraction force following sustained muscular activity [1]. This fatigue can arise from various factors, such as the mode and duration of exercise, age, gender, and health status [2]. It has been shown that exercise-induced muscle fatigue can detract from athletes’ sports performance [3], including muscle strength and power.

Currently, athletes frequently utilize compression garments to minimize the development of exercise-induced muscle fatigue during or after various training and competition sessions. Wearing compression garments during or after exercise can improve venous return flow, thereby reducing muscle fatigue, and enhancing muscle recovery [4]. However, the effect of compression garments on strength performance has been inconsistent across studies. Négyesi et al. [5] found that wearing below-knee compression garments during exercise reduces the loss of maximal voluntary contraction power in the lower limbs post-exercise. In addition, Jakeman et al. [6] showed that young women who wore compression garments immediately after deep jump training significantly reduced the loss of maximum strength in reverse longitudinal jump, squat jump, and knee extension. Blumkaitis et al. [7] found that healthy men who wore calf tights for 30 min of recovery immediately after completing 100 repetitions of deep jump training were better able to maintain the relative mean contraction strength and mean power of the squat jump at 24 h and 48 h.

Several potential mechanisms may explain how compression garments can mitigate exercise-induced muscle fatigue. First, compression garments enhance muscle pump function, thereby improving venous return and blood circulation within the muscles. This, in turn, facilitates more efficient oxygen utilization by the muscles [5]. Additionally, compression garments have been shown to reduce muscle swelling, alleviate soreness, and attenuate the inflammatory response following exercise [7]. Furthermore, wearing compression garments during physical activity helps stabilize the muscles and surrounding tissues, minimizing excessive movement and enhancing movement efficiency [6].

However, some studies have concluded that wearing compression garments during or after exercise does not alleviate the loss of muscle strength and power. Mizuno et al. [8] demonstrated that compression garments did not mitigate the extent of the loss of maximal voluntary contraction strength in reverse vertical jump and knee extension after 120 min of uphill running while wearing compression garments. Brown et al. [9] found that men with no experience in resistance training who wore compression garments for 72 h of recovery immediately after centrifugal exercise did not promote faster recovery of strength. Therefore, the effect of compression garments on the post-exercise recovery of strength performance remains inconclusive.

Previous meta-analyses have demonstrated the beneficial effects of compression garments in recovering from exercise-induced muscle damage [10,11,12]. However, many studies have overlooked factors such as the participants’ training experience and the specific body areas covered by the compression garments. Additionally, previous meta-analyses included a limited number of studies, and certain experimental groups may have been confounded by other interventions, or control groups may have also received interventions, thereby affecting the final results. In this study, we analyzed the effects based on the areas covered by the compression garments to provide a clearer understanding of how compression garments impact different body regions. Moreover, a subgroup analysis was conducted based on the participants’ training experience to assess how compression garments may affect individuals with varying levels of training.

## 2. Materials and Methods

### 2.1. Design

This systematic review and meta-analysis followed the standards and recommendations of the Preferred Reporting Items for Systematic Reviews and Meta-Analyses (PRISMA, 2020) [13]. The protocol was registered with PROSPERO (CRD42024517916).

### 2.2. Search Strategy

The PubMed, Web of Science, EBSCO, Cochrane, and Scopus databases were searched for all studies on the effects of compression garments on muscle strength and power after exercise-induced muscle fatigue, up to 20 October 2024, using the following keywords and MESH terms: compression garment, muscle strength, and power (Table S1). Additionally, we hand-searched the reference lists of all identified systematic reviews and meta-analyses to find any other relevant studies.

### 2.3. Eligibility Criteria

The Population, Intervention, Comparison, Outcome (PICO) framework was used to define the inclusion criteria: (a) Population: adults; (b) Intervention: randomized controlled trials (RCTs) with subjects randomly assigned to either compression garments or control group, where compression garments were worn during or after exercise (including running, drop jump, resistance training, and other forms of physical activity), while the control group receives no recovery interventions before, during, or after exercise; (c) Comparison: measured muscle strength or power with baseline, comparing results at 0 h, 24 h (1–24 h), 48 h (25–48 h), 72 h (49–72 h), or over 72 h (>72 h); (d) Outcome: the primary outcomes were muscle strength and power. Muscle strength was measured using the maximal voluntary contraction (MVC), whereas power was measured using the countermovement jump (CMJ) or squat jump (SJ).

Exclusion criteria: (a) compression garments not worn within 2 h after exercise; (b) multiple interventions in experimental group; (c) control group engaged in recovery-enhancing exercise; and (d) non-English publications.

### 2.4. Data Extraction

The data extraction process was independently conducted by two authors (X.L. and L.D.), encompassing author information, subject characteristics (n, gender, and training experience), types of compression garments, the nature and intensity of fatigue training, rest intervals between training and testing, as well as data on muscle strength and power [mean and standard deviation (SD)]. In the case of disagreement between the two authors, a third author (L.Y.) was consulted to facilitate a consensus.

### 2.5. Methodological Quality Assessment

The Cochrane collaboration tool, including items of selection bias, performance bias, detection bias, attrition bias, reporting bias, and other biases, was used to evaluate the quality of eligible studies. Based on the responses to the signaling questions, each item was categorized as either “low risk”, “unclear risk”, or “high risk” [14].

### 2.6. Sensitivity Analysis

Sensitivity analysis was conducted to assess the robustness of meta-analytic results and identify any individual studies that might disproportionately influence the overall effect estimates. Each study included in the meta-analysis was individually removed from the dataset. After each study was removed, the overall effect size and its 95% confidence interval (CI) were recalculated using the remaining studies. This process was repeated for each study in the dataset to systematically assess the impact of each individual study on the overall estimate.

### 2.7. Statistical Analysis

Given that the included studies explored the effect of compression garments on muscle strength and power across multiple rest intervals, it cannot be assumed that the results are independent across rest intervals. Statistical analyses in this study were based on a three-level restricted maximum likelihood random effects model, using the metafor for R package [15]. The computational approach used in a previous study was adopted [16]. This model takes into account the relevance of within-study effect sizes by providing variance estimates within studies (level 2) and between studies (level 3) [15]. The primary outcome metric in this study was expressed as “mean ± SD”, with the Hedges’s g value representing the difference between the mean of the compression garments group and the control group, divided by the pooled standard deviation of the number of individuals of interest. The total effect size (ES) was assessed based on the Hedges’s g values classification, where effect sizes were defined as <0.40 for small, 0.40–0.70 for moderate, and >0.70 for large [17]. Heterogeneity was assessed by *I*^2^, where *I*^2^ < 25%, 25% < *I*^2^ < 50%, 50% < *I*^2^ < 75%, and *I*^2^ > 75% indicate no heterogeneity, low heterogeneity, moderate heterogeneity, and high heterogeneity, respectively [14].

In subgroup analyses, we used rest periods (0 h, 1–24 h, 25–48 h, 49–72 h, and over 72 h), test site (upper and lower limbs), and training experience (with and without training experience) to explore the effects of compression garments on muscle strength and power following exercise-induced muscle fatigue. Specifically, participants were categorized as “trained” if they had prior experience in resistance training, typically defined as engaging in regular strength training sessions for a minimum duration of several months. Conversely, participants were classified as “untrained” if they had no or minimal experience in structured resistance training. All data analyses were performed using R4.2.2 (R Foundation for Statistical Computing, Vienna, Austria) and Comprehensive Meta-Analysis 3.0 (CMA, Englewood, CO, USA).

## 3. Results

### 3.1. Study Selection

**Figure 1:**
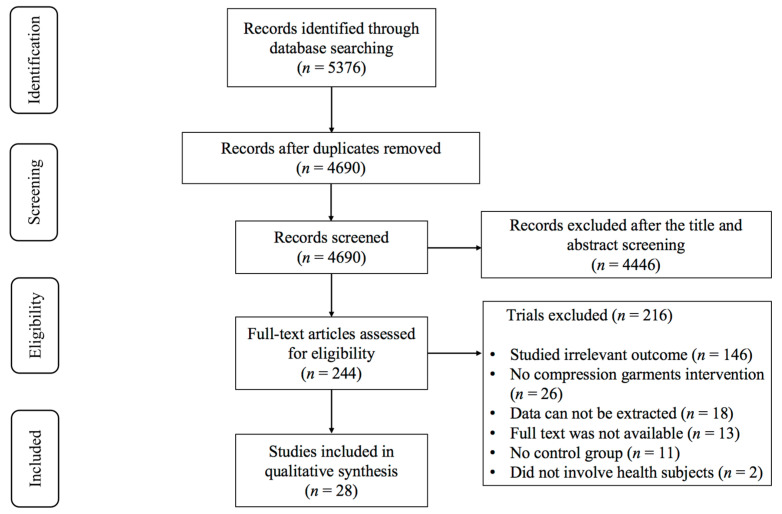
PRISMA flowchart of study selection.

Two authors (X.L. and L.D.) conducted an independent review of all articles’ titles, abstracts, and full texts to identify studies examining the effects of compression garments on muscle strength and power after exercise-induced muscle fatigue. The screening process adhered to strict inclusion and exclusion criteria. Among the 5376 articles identified, a total of 28 studies [5,18,19,20,21,22,23,24,25,26,27,28,29,30,31,32,33,34,35,36,37,38,39,40,41,42,43,44] were considered eligible for meta-analysis (Figure 1).

### 3.2. Characteristics of the Included Studies

The main characteristics of the participants and the interventions were shown in Table S2. The included studies encompassed a total of 528 participants, with an average age ranging from 20 to 43 years. A total of twenty-five studies focused on participants with training experience [5,18,19,20,21,22,23,24,25,28,29,30,31,32,33,34,36,37,38,39,40,41,42,43,44], while three studies focused on participants without training experience [26,27,35]. Categorizing the included studies based on the location of compression garment use, three studies used upper limb compression garments [25,26,27], and twenty-five studies used lower limb compression garments [5,18,19,20,21,22,23,24,28,29,30,31,32,33,34,35,36,37,38,39,40,41,42,43,44]. The rest interval between fatigue exercise and testing ranged from 0 to 96 h. Regarding the test results, fifteen studies used CMJ as a test outcome [18,19,20,21,22,23,29,30,32,34,35,37,39,41,44], fifteen studies used MVC as a test outcome [5,22,24,25,26,27,28,31,33,35,36,38,40,42,43], and one study used SJ as a test outcome [35].

### 3.3. Main Effect

Overall, the 28 included studies reported 107 effect sizes for meta-analysis. Compression garments had significant restorative effects on muscle strength (Hedges’s g = −0.28, 95% CI: −0.38 to −0.18, *p* < 0.01, *I*^2^ = 82.87%, Figure S1, and Table 1) and power after exercise-induced muscle fatigue (Hedges’s g = −0.23, 95% CI: −0.34 to −0.11, *p* < 0.01, *I*^2^ = 80.09%, Figure S2 and Table 2). To further explore the role of compression garments in mitigating the decline in muscle strength and power caused by exercise fatigue, subgroup analyses were conducted.

### 3.4. Subgroup Analysis

When the rest intervals were 1–24 h (Hedges’s g = −0.26, 95% CI: −0.38 to −0.13, *p* < 0.01, *I*^2^ = 74.52%), 25–48 h (Hedges’s g = −0.25, 95% CI: −0.45 to −0.06, *p* = 0.01, *I*^2^ = 77.58%), and over 72 h (Hedges’s g = −0.76, 95% CI: −1.04 to −0.48, *p* < 0.01, *I*^2^ = 0%), compression garments had a significant effect on mitigating the decline in muscle strength after exercise-induced muscle fatigue. However, when the rest intervals were 0 h (Hedges’s g = −0.23, 95% CI: −0.48 to 0.01, *p* = 0.06, *I*^2^ = 90.08%) and 49–72 h (Hedges’s g = −0.34, 95% CI: −0.71 to 0.03, *p* = 0.07, *I*^2^ = 87.57%), the compression garments did not significantly mitigate the decline of muscle strength after exercise-induced muscle fatigue (Table 1).

In addition, lower limb compression garments significantly mitigated the decline in muscle strength after exercise-induced muscle fatigue (Hedges’s g = −0.30, 95% CI: −0.41 to −0.19, *p* < 0.01, *I*^2^ = 85.05%). Conversely, upper limb compression garments did not significantly mitigate the decline of muscle strength after exercise-induced muscle fatigue (Hedges’s g = −0.11, 95% CI: −0.23 to 0.01, *p* = 0.07, *I*^2^ = 39.89%, Table 1).

In addition, when the rest interval was over 72 h (Hedges’s g = −0.91, 95% CI: −1.58 to −0.24, *p* < 0.01, *I*^2^ = 0%), upper limb compression garments had a significant effect on mitigating the decline in muscle strength after exercise-induced muscle fatigue. For rest intervals that were 1–24 h (Hedges’s g = −0.05, 95% CI: −0.24 to 0.15, *p* = 0.63, *I*^2^ = 49.47%), 25–48 h (Hedges’s g = −0.12, 95% CI: −0.31 to 0.08, *p* = 0.24, *I*^2^ = 0.00%), and 49–72 h (Hedges’s g = −0.20, 95% CI: −0.76 to 0.36, *p* = 0.48, *I*^2^ = 60.90%), upper limb compression garments did not significantly mitigate the decline in muscle strength after exercise-induced muscle fatigue. In comparison, for the same rest intervals (1–24 h, (Hedges’s g = −0.28, 95% CI: −0.42 to −0.15, *p* < 0.01, *I*^2^ = 76.50%), 25–48 h (Hedges’s g = −0.31, 95% CI: −0.58 to −0.05, *p* = 0.02, *I*^2^ = 84.98%), and over 72 h, (Hedges’s g = −0.73, 95% CI: −1.04 to −0.42, *p* < 0.01, *I*^2^ = 35.59%)), lower limb compression garments significantly mitigated the decline in muscle strength after exercise-induced muscle fatigue. When the rest intervals were 0 h (Hedges’s g = −0.23, 95% CI: −0.48 to 0.02, *p* = 0.07, *I*^2^ = 90.08%) and 49–72 h (Hedges’s g = −0.42, 95% CI: −0.91 to 0.08, *p* = 0.10, *I*^2^ = 92.00%), compression garments did not mitigate the decline in muscle strength after exercise-induced muscle fatigue (Table 1).

Furthermore, compression garments significantly mitigated the decline in muscle strength in the trained (Hedges’s g = −0.26, 95% CI: −0.37 to −0.15, *p* < 0.01, *I*^2^ = 84.47%) and untrained individuals (Hedges’s g = −0.37, 95% CI: −0.57 to −0.16, *p* < 0.01, *I*^2^ = 68.16%) after exercise-induced muscle fatigue. Specifically, the effectiveness of compression garments was more pronounced in trained individuals compared to untrained ones (Table 1).

Moreover, for trained individuals, compression garments significantly mitigated the decline in muscle strength during rest intervals of 1–24 h (Hedges’s g = −0.28, 95% CI: −0.42 to −0.14, *p* < 0.01, *I*^2^ = 77.11%) and over 72 h (Hedges’s g = −1.02, 95% CI: −1.57 to −0.46, *p* < 0.01, *I*^2^ = 0%) after exercise-induced muscle fatigue. However, during rest intervals of 0 h (Hedges’s g = −0.23, 95% CI: −0.48 to 0.02, *p* = 0.07, *I*^2^ = 90.08%), 25–48 h (Hedges’s g = −0.18, 95% CI: −0.38 to 0.02, *p* = 0.08, *I*^2^ = 75.58%), and 49–72 h (Hedges’s g = −0.34, 95% CI: −0.89 to 0.21, *p* = 0.23, *I*^2^ = 92.51%), compression garments did not significantly mitigate the decline in muscle strength after exercise-induced muscle fatigue. For untrained individuals, compression garments significantly mitigated the reduction in muscle strength during the rest interval of over 72 h (Hedges’s g = −0.67, 95% CI: −0.99 to −0.34, *p* < 0.01, *I*^2^ = 0%) after exercise-induced muscle fatigue. However, during rest intervals of 1–24 h (Hedges’s g = −0.17, 95% CI: −0.45 to 0.11, *p* = 0.23, *I*^2^ = 60.74%), 25–48 h (Hedges’s g = −0.51, 95% CI: −1.05 to 0.03, *p* = 0.07, *I*^2^ = 78.21%), and 49–72 h (Hedges’s g = −0.36, 95% CI: −0.84 to 0.11, *p* = 0.13, *I*^2^ = 72.25%) compression garments did not significantly mitigate the decline in muscle strength after exercise-induced muscle fatigue (Table 1).

Finally, when the rest interval was 1–24 h (Hedges’s g = −0.27, 95% CI: −0.42 to −0.11, *p* < 0.01, *I*^2^ = 61.52%), compression garments had a significant effect on mitigating the decline in power after exercise-induced muscle fatigue. However, for rest intervals of 0 h (Hedges’s g = −0.11, 95% CI: −0.33 to 0.12, *p* = 0.34, *I*^2^ = 85.45%), 25–48 h (Hedges’s g = −0.30, 95% CI: −0.61 to 0.001, *p* = 0.05, *I*^2^ = 85.38%), 49–72 h (Hedges’s g = −0.38, 95% CI: −0.77 to 0.004, *p* = 0.05, *I*^2^ = 68.86%), and over 72 h (Hedges’s g = −0.21, 95% CI: −0.72 to 0.29, *p* = 0.41, *I*^2^ = 74.23%), compression garments did not significantly mitigate the decline in power after exercise-induced muscle fatigue (Table 2).

### 3.5. Meta-Regression

Meta-regression analyses showed no significant associations between rest intervals (*p* = 0.236), body parts (*p* = 0.690), training experience (*p* = 0.672), rest intervals for the upper limb (*p* = 0.597), rest intervals for the lower limb (*p* = 0.222), body parts for trained individuals (*p* = 0.777), or body parts for trained individuals (*p* = 0.983) and muscle strength. Additionally, no significant association was found between rest intervals and power (*p* = 0.591, Table S3).

### 3.6. Risk of Bias

**Figure 2:**
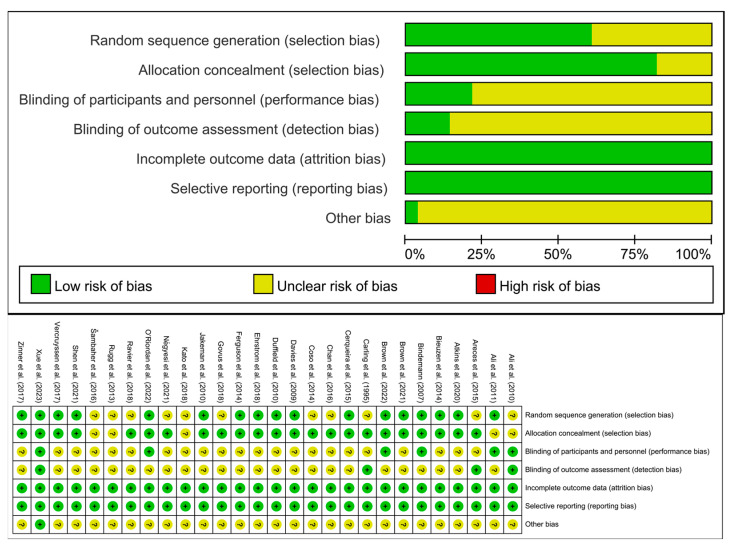
Results of Cochrane risk of bias tool [5,18,19,20,21,22,23,24,25,26,27,28,29,30,31,32,33,34,35,36,37,38,39,40,41,42,43,44].

The methodological quality of the included studies was assessed using the Cochrane Risk Assessment Tool, which focuses on six biases: selection, performance, detection, attrition, reporting, and others, resulting in a classification of the included studies into high, moderate, and low quality (Figure 2). Specifically, one study [43] was assessed as having a low risk of bias, while twenty-seven studies [5,18,19,20,21,22,23,24,25,26,27,28,29,30,31,32,33,34,35,36,37,38,39,40,41,42,44] were categorized as having a moderate risk. The primary issue with studies at moderate risk of bias is that neither the participants nor the researchers were blinded during the group assignment process. When either the participants or the researchers are aware of the group allocation, it can lead to psychological or behavioral changes that affect the reliability and accuracy of the experimental results. Additionally, studies at moderate risk of bias did not implement blinding for outcome assessment or the experimental process, which may result in researchers’ subjective judgments influencing the objectivity of the results.

### 3.7. Publication Bias

A potential publication bias was assessed by examining the funnel plot (Figure S3), which revealed no asymmetry. The Egger’s test results showed that a small sample size had no significant impact on the final result of power (*p* = 0.127; Table S2). However, the Egger’s test results show that a small sample size may have a significant impact on the final result of strength (*p* < 0.001; Table S2). The inclusion of 15 virtual studies through the Trim and Fill analysis did not alter the results, suggesting that the findings of the strength are robust (Table S3).

### 3.8. Sensitivity Analyses

The sensitivity analysis revealed no alternation in the direction or magnitude of the overall effect of compression garments on muscle strength (Figure S4) and power (Figure S5) after exercise-induced muscle fatigue, regardless of the exclusion of any of the included studies.

## 4. Discussion

### 4.1. Effects of Compression Garments on Muscle Strength After Exercise-Induced Muscle Fatigue

The use of compression garments after exercise-induced muscle fatigue aids in promoting the recovery of muscle strength, potentially due to the following reasons. Firstly, compression garments enhance muscle tissue recovery [45]. When worn after exercise-induced muscle fatigue, they reduce muscle inflammation [11], thereby alleviating post-exercise muscle soreness [6]. Furthermore, compression garments enhance muscle pump function [46], improving peripheral blood circulation and venous return [4,46,47], which may facilitate nutrient delivery to muscle tissues, eliminate muscle damage markers [11], and reduce muscle swelling [48]. Secondly, compression garments immobilize muscles and other subcutaneous tissues during exercise, minimizing swaying and improving exercise economy [49]. Finally, the enhanced recovery associated with wearing compression garments may also be influenced psychologically [38], as experiments involving compression garments cannot be blinded, leading to positive psychological cues during training and recovery. Duffield et al. [50] has found that compression garments significantly improve participants’ subjective recovery level.

While the findings of this study aligned with numerous studies, there are also studies with contrasting results. A meta-analysis by Négyesi et al. [51] concluded that compression garments did not mitigate the decline in strength performance after exercise. Similarly, Mizuno et al. [8] found that wearing compression garments during and after exercise did not significantly mitigate the decline in muscle strength. There are several reasons why compression garments fail to exert a significant recovery effect after exercise-induced muscle fatigue. Firstly, it is possible that the intensity of the exercise causing muscle fatigue was too low, resulting in minimal muscle fatigue. Therefore, compression garments could not achieve a recovery effect. Secondly, training intensity mainly affects muscle pumping [46], which can lead to lower venous return when the intensity is too low, diminishing the effectiveness of compression garments. Additionally, different types of compression garments require varying pressure levels to achieve the best results [8,52], and the recovery effect can be affected when the pressure is too low. Lastly, anthropometric variations among participants, such as height, weight, and arm circumference [8,51], can lead to discrepancies in the compression achieved by the same garments, potentially affecting their effectiveness.

### 4.2. Subgroup Analysis

Different parts of the compression garments may exert varying effects on mitigating the decline of muscle strength after exercise-induced muscle fatigue [52]. In this study, we compared the effects of wearing compression garments on the upper and lower limbs. Our findings indicated that lower limb compression garments significantly mitigated the decline in muscle strength after exercise-induced muscle fatigue, whereas upper limb compression garments did not yield a significant recovery effect. Numerous studies have demonstrated that compression garments for the lower limbs can foster the recovery of muscle strength. O’Riordan et al. [37] found that wearing lower limb compression garments for 4 h after completing lower body eccentric resistance training significantly improved the recovery effects of CMJ and isometric mid-thigh pull (IMTP). Ravier et al. [38] indicated that wearing lower limb compression tights during sport-specific training significantly reduced the decline in MVC and the rate of force development (RFD) in handball players. However, the effects on compression garments on the upper limbs in enhancing recovery remains uncertain. The insignificant reduction in the magnitude of decline in muscle strength after exercise-induced muscle fatigue when wearing upper limb compression garments may be attributed to the area covered by the garments and the size of the muscles. It has been postulated that compression garments can enhance athletic performance by activating the proprioception of the limb through limb pressurizing [53]. Most compression garments designed for the upper limb cover the entire upper limb or merely the forearm, which is less effective in enhancing proprioception compared to lower limb compression garments that cover a smaller skin area. Furthermore, although external compression garments for the limbs have been experimentally proven to significantly enhance limb blood flow [46], the cross-sectional area of the lower limb muscles is generally much larger than that of the upper limb muscles. Consequently, the vascular density in the upper limb muscles is lower than in the lower limb muscles, and the primary mechanism for compression garments is closely associated with changes in venous and muscular blood flow [4]. Therefore, upper limb compression garments did not significantly mitigate the decline in muscle strength after exercise-induced muscle fatigue.

Considering the influence of the body part on which compression garments are worn, we next determined whether rest intervals could affect the muscle strength when wearing upper limb and lower limb compression garments. Our results showed that lower limb compression garments significantly mitigated the decline in muscle strength after exercise-induced muscle fatigue when the rest intervals were 1–24 h and over 72 h. Conversely, upper limb compression garments significantly mitigated the decline in muscle strength after exercise-induced muscle fatigue when the rest interval was over 72 h. Ravie et al. [38] found that wearing lower limb compression garments during handball training mitigated the decline in muscle strength immediately post-exercise. In addition, Bieuzen et al. [22] found that wearing lower limb compression garments favored muscle strength recovery 1 h and 24 h after trail running. Potential explanations for these results may be as follows: (a) Wearing compression garments during exercise reduces muscle sway, enhancing the exercise economy, thus reducing energy expenditure during exercise. (b) Wearing compression garments during or after exercise improves muscle pump function, promoting blood circulation and venous return flow to the limb muscles, which is beneficial for reducing muscle soreness and swelling. (c) The present study primarily included studies where compression garments were worn during exercise and within 24 h post-exercise. Therefore, when the rest intervals were 1–24 h, compression garments significantly mitigated the decline in muscle strength after exercise-induced muscle fatigue. (d) Delayed onset muscle soreness occurs following fatigue training and is inversely related to athletic performance. A greater severity of DOMS results in prolonged muscle recovery and reduced muscle strength. Therefore, when the rest interval is 25–48 h, wearing compression garments during or after exercise significantly mitigates the decline in muscle strength following exercise-induced muscle fatigue. (e) Following high-intensity exercise, there is a significant consumption of energy substances in the muscles, accompanied by a significant increase in hydrogen ions, creatine kinase, and other indicators of muscle damage. However, after resting for over 72 h, the energy material in the muscle is fully replenished, and the muscle damage markers produced during exercise are fully metabolized and cleared. Additionally, upper limbs typically have smaller muscle mass and different vascular architecture compared to lower limbs, which could affect the distribution of pressure and the subsequent recovery benefits. Furthermore, compression garments can enhance muscle pump function, and improve venous return and blood flow in the muscles, thereby increasing the muscles’ ability to utilize oxygen from the blood and facilitating nutrient transport. Consequently, resting for over 72 h has a significant positive effect.

In this study, subgroup analysis was also conducted based on the training experience of the participants, revealing that compression garments significantly mitigated the decline in muscle strength in the trained and untrained individuals after exercise-induced muscle fatigue. Moreover, the effectiveness of compression garments was more pronounced in trained individuals compared to untrained ones. Brown et al. [25] and Jakeman et al. [35] found that both trained and untrained individuals exhibited a reduced effect of exercise-induced muscle fatigue on muscle strength when wearing compression garments. In the group with training experience, prior training altered the excitability of spinal motoneurons and induced synaptic production in the spinal cord, resulting in the formation of muscle memories [54], which facilitates muscle recovery. Additionally, muscle memory is influenced by the myonuclear number [55]. Since trained individuals possess greater muscle mass compared to untrained individuals, their muscle recovery is faster. Therefore, the trained individuals exhibited better recovery after wearing compression garments compared to the untrained individuals.

Considering the influence of training experience, we next determined whether rest intervals could affect the muscle strength in trained and untrained individuals. Our results showed that compression garments significantly mitigated the decline in muscle strength, during rest intervals of 1–24 h for trained individuals and over 72 h for both trained and untrained individuals, after exercise-induced muscle fatigue. Goto et al. [56] found that compression garments significantly improved the recovery of muscle strength 24 h after exercise in trained participants. Similarly, Cerqueira et al. [27] found that compression garments significantly mitigated the decline in muscle strength after exercise-induced muscle fatigue in untrained participants. The reason why wearing compression garments during a 1–24 h rest interval in trained participants improves the recovery of muscle strength after exercise-induced muscle fatigue is not solely attributed to the benefits of compression garments, but also because trained participants possess relevant training experience, which contributes to the formation of muscle memories and enhances the muscle’s ability for recovery.

### 4.3. Effects of Compression Garments on Power After Exercise-Induced Muscle Fatigue

This study found that compression garments significantly mitigated the decline in power after exercise-induced muscle fatigue, which is consistent with the findings of previous studies [11,39], showing that wearing compression garments after exercise-induced muscle fatigue can mitigate the decline in power. The potential reasons are that (a) compression garments can alleviate the inflammatory response in muscles during exercise-induced muscle fatigue [11], thereby reducing post-exercise muscle soreness [6]; (b) compression garments immobilize subcutaneous tissues, including muscles, during exercise-induced muscle fatigue, minimizing muscle sway and enhancing neural inputs during recovery [48]; (c) wearing compression garments can increase or maintain body surface temperature compared to regular sportswear, and maintaining body temperature facilitates higher muscle power output during exercise [57]; and (d) compression garments activate proprioception in the limbs through pressure, improving athletic performance [53].

However, the efficacy of compression garments on power recovery varies depending on the rest interval. Subgroup analysis showed that when the rest interval was 1–24 h, compression garments had a significant effect on mitigating the decline in power after exercise-induced muscle fatigue, which is consistent with the results of previous studies. Hill et al. [58] found that wearing high-pressure compression garments 24 h post-exercise significantly mitigated the decline in CMJ caused by exercise-induced muscle fatigue. In addition, Mizuno et al. [59] reported that after wearing lower limb compression garments for 24 h following endurance exercise, the CMJ height was notably higher compared to the control group, indicating that compression garments can expedite recovery from exercise-induced muscle fatigue.

While the findings of this study support the notion that wearing compression garments after exercise-induced muscle fatigue reduces power decrement, it has also been proposed that wearing compression garments does not mitigate the adverse effects of such fatigue on power. Davies et al. [29] showed that wearing compression garments immediately after deep jumps training for 48 h did not mitigate the decline in the reverse longitudinal jump performance. The ineffectiveness of compression garments in promoting power recovery may stem from the need to combine different types of compression garments with varying pressure levels to optimal results [8,52]. Specifically, too low of a pressure in the compression garments can hinder the recovery effect. Notably, Davies et al. [29] did not report the pressure level of the compression garments, which might explain the non-significant findings.

### 4.4. Strength and Limitations

Our study introduces several strengths to the existing body of research on compression garments and their effects on exercise-induced muscle fatigue. First, our comprehensive meta-analysis includes a larger number of studies and participants than previous reviews, providing a more robust and reliable assessment of the overall effects. Additionally, we conducted subgroup analyses based on rest intervals, body parts, and training experience, which allowed us to identify specific conditions under which compression garments may be most effective. This level of detail enhances the applicability of our findings to diverse populations and training contexts.

This meta-analysis also had some potential limitations. First, many of the included studies did not measure or report specific pressure values for the compression garments, thus we did not delve deeply into the effects of different compression garments pressure levels on muscle strength and power after exercise-induced muscle fatigue. For future research, we recommend standardizing or at least reporting compression garment pressure and fit more comprehensively. Selecting compression garments of varying sizes based on limb circumference could ensure optimal pressure, thereby facilitating effective recovery. Second, the majority of participants in the included studies were predominantly male, which limits the applicability of our results to female athletes or older populations. This demographic skew precluded us from conducting gender-specific subgroup analyses and may introduce bias in interpreting the effects of compression garments across different populations. Future research should prioritize recruiting more diverse cohorts, including female athletes and older individuals, to better understand the potential benefits and limitations of compression garments in these underrepresented groups. Third, the included studies involved compression garments interventions, which were not fully blinded. This limitation may introduce bias, as participants aware of the intervention could experience positive psychological cues, which might inflate the observed effects. To address this concern, future studies should consider employing strategies to minimize such biases. For instance, using sham garments that are indistinguishable from actual compression garments could help mitigate the placebo effect. Additionally, measuring and reporting participants’ expectancy effects would provide valuable insights into the extent to which psychological factors influence the outcomes. Although blinding is challenging in studies involving compression garments, these strategies could enhance the robustness of the findings and improve the interpretation of results. Lastly, the uneven distribution of data points across the rest intervals may impact the precision of our subgroup analyses. Specifically, some intervals contain relatively few data points, which could result in wider confidence intervals and greater uncertainty in the estimated effects of compression garments on muscle strength and power within those intervals. This uneven distribution limits the robustness of our conclusions for certain time frames and highlights the need for more comprehensive data collection across a broader range of rest periods in future studies.

### 4.5. Practical Implications

This study underscores the potential role of compression garments in mitigating muscle fatigue following exercise. We recommend that athletes and exercise enthusiasts consider wearing compression garments either during or after exercise, as they have demonstrated significant recovery benefits. For athletes participating in multiple competitions over consecutive days, wearing compression garments (e.g., shirts, shorts, or sleeves) during or after each event can help reduce the impact of exercise-induced muscle fatigue on subsequent performance. The choice of garment and timing of use should be tailored to the specific demands of the sport and individual preferences. Compression garments can be incorporated into routine training and post-training protocols to enhance recovery and minimize the accumulation of muscle fatigue. This may be particularly beneficial for individuals engaged in high-intensity or prolonged exercise sessions, where muscle fatigue can significantly affect performance.

## 5. Conclusions

Our study demonstrates that compression garments significantly mitigated muscle strength decline following exercise-induced muscle fatigue, offering valuable insights for recovery strategies. For upper limbs, benefits were most notable after resting for over 72 h, while lower limbs showed improvements during shorter (1–24 h) and longer (over 72 h) rest intervals. This suggests that the effectiveness of compression garments may vary by muscle group, likely due to differences in muscle mass and vascular architecture. Both trained and untrained individuals benefited, with trained individuals experiencing more pronounced effects, possibly due to their higher baseline fitness. Compression garments also significantly mitigated power decline during the 1–24-h rest interval. These findings highlight the potential of compression garments as a versatile recovery tool. Future research should explore optimal compression levels for different muscle groups and include more diverse populations to enhance generalizability.

## Figures and Tables

**Table 1:** Results of moderator analysis of strength.

Moderator	Hedges’s g (95% CI)	*I*^2^ (%)	*p* Value
Overall	−0.27 (−0.22, −0.14)	82.87	<0.01
Rest intervals			
0 h	−0.23 (−0.48, 0.02)	90.08	0.07
0 h < t ≤ 24 h	−0.26 (−0.38, −0.13)	74.52	<0.01
24 h < t ≤ 48 h	−0.25 (−0.45, −0.06)	77.58	0.01
48 h < t ≤ 72 h	−0.34 (−0.71, 0.03)	85.57	0.07
t > 72 h	−0.76 (−1.04, −0.48)	0.00	<0.01
Body parts			
Upper limb	−0.19 (−0.23, 0.01)	39.89	0.07
Lower limb	−0.30 (−0.41, −0.19)	85.05	<0.01
Training experience			
Trained individuals	−0.26 (−0.37, −0.15)	84.47	<0.01
Untrained individuals	−0.37 (−0.57, −0.16)	68.16	<0.01
Rest intervals at different body parts			
Upper limb			
0 h < t ≤ 24 h	−0.05 (−0.24, 0.15)	49.47	0.63
24 h < t ≤ 48 h	−0.12 (−0.31, 0.08)	0.00	0.24
48 h < t ≤ 72 h	−0.20 (−0.76, 0.36)	60.90	0.48
t > 72 h	−0.91 (−1.58, −0.24)	0.00	0.01
Lower limb			
0 h	−0.23 (−0.48, 0.02)	90.08	0.22
0 h < t ≤ 24 h	−0.28 (−0.42, −0.15)	76.50	<0.01
24 h < t ≤ 48 h	−0.31 (−0.58, 0.05)	84.98	<0.01
48 h < t ≤ 72 h	−0.42 (−0.91, 0.08)	92.00	0.10
t > 72 h	−0.73 (−1.04, −0.42)	35.59	<0.01
Rest intervals at different training experience			
Trained individuals			
0 h	−0.23 (−0.48, 0.02)	90.08	0.07
0 h < t ≤ 24 h	−0.28 (−0.42, −0.14)	77.11	<0.01
24 h < t ≤ 48 h	−0.18 (−0.38, 0.02)	75.58	0.08
48 h < t ≤ 72 h	0.34 (−0.89, 0.21)	92.51	0.23
t > 72 h	−1.02 (−1.57, −0.46)	0.00	<0.01
Untrained individuals			
0 h < t ≤ 24 h	−0.17 (−0.45, 0.11)	60.74	0.23
24 h < t ≤ 48 h	−0.51 (−1.05, 0.03)	78.21	0.07
48 h < t ≤ 72 h	−0.36 (−0.84, 0.11)	72.25	0.13
t > 72 h	−0.67 (−0.99, −0.34)	0.00	<0.01

Abbreviations: CI, confidence interval.

**Table 2:** Results of moderator analysis of power.

Moderator	Hedges’s g (95% CI)	*I*^2^ (%)	*p* Value
Overall	−0.23 (−0.34, −0.11)	80.09	<0.01
Rest intervals			
0 h	−0.11 (−0.33, 0.12)	85.45	0.34
0 h < t ≤ 24 h	−0.27 (−0.42, −0.11)	61.52	<0.01
24 h < t ≤ 48 h	−0.30 (−0.61, 0.001)	85.38	0.05
48 h < t ≤ 72 h	−0.38 (−0.77, 0.004)	68.86	0.05
t > 72 h	−0.21 (−0.72, 0.29)	74.23	0.41

Abbreviations: CI, confidence interval.

## Data Availability

All data generated or analyzed during this study are included in the article/Supplementary Materials.

## Supplementary Materials

The following supporting information can be downloaded at https://www.mdpi.com/article/10.3390/life15030438/s1: Figure S1: Meta-analysis results of the effects of compression garments on muscle strength after exercise-induced muscle fatigue; Figure S2: Meta-analysis results of the effects of effects of compression garments on power after exercise-induced muscle fatigue; Figure S3: Funnel plots; Figure S4: Sensitivity analyses results of muscle strength; Figure S5: Sensitivity analyses results of power; Table S1: Search terms for compression garment, muscle strength, and power; Table S2: Characteristics of the studies included in this meta-analysis; Table S3: Results of meta-regression; Table S4: Results of Egger’s test; Table S5: Results of trim and fill analysis.
