# Supplementary material for: Effects of Compression Garments on Muscle Strength and Power Recovery Post-Exercise: A Systematic Review and Meta-Analysis
Source: Life (Basel). 2025 Mar 11;15(3):438. doi: 10.3390/life15030438 (PMC11944185; doi:10.3390/life15030438)
Supplement: Supplementary file 1 [file life-15-00438-s001.zip › life-3496128-supplementary.pdf]

Supplemental material

Effects of compression garments on muscle strength and power recovery post-exercise: a systematic review and meta-analysis

Figure S1 Meta-analysis results of the effects of compression garments on muscle strength after exercise-induced muscle fatigue........................ 2
Figure S2 Meta-analysis results of the effects of effects of compression garments on power after exercise-induced muscle fatigue........................3
Figure S3 Funnel plots.. 4
Figure S4 Sensitivity analyses results of muscle strength........................ 5
Figure S5 Sensitivity analyses results of power.......................................6
Table S1 Search terms for compression garment, muscle strength, and power.. 7
Table S2 Characteristics of the studies included in this meta-analysis......8
Table S3 Results of meta-regression...18
Table S4 Results of Egger's test...19
Table S5 Results of trim and fill analysis.. 20

Figure S1 Meta-analysis results of the effects of compression garments on muscle strength after exercise-induced muscle fatigue

Model	Study name	Hedges's g	Standard error	Variance	Lower limit	Upper limit	Z-Value	p-Value
	Brown et al. (2021)A1	0.000	0.182	0.033	-0.356	0.356	0.000	1.000
	Brown et al. (2021)A2	-0.009	0.182	0.033	-0.366	0.347	-0.050	0.960
	Brown et al. (2021)A3	-0.098	0.182	0.033	-0.455	0.259	-0.538	0.590
	Brown et al. (2022)A1	-0.537	0.138	0.019	-0.808	-0.267	-3.890	0.000
	Brown et al. (2022)A2	-0.479	0.137	0.019	-0.748	-0.210	-3.492	0.000
	Brown et al. (2022)A3	-0.435	0.136	0.019	-0.702	-0.167	-3.185	0.001
	Brown et al. (2022)B1	-0.300	0.135	0.018	-0.564	-0.035	-2.222	0.026
	Brown et al. (2022)B2	-0.203	0.134	0.018	-0.465	0.060	-1.513	0.130
	Brown et al. (2022)B3	-0.009	0.133	0.018	-0.270	0.252	-0.067	0.946
	Carling et al. (1995)1	-0.061	0.174	0.030	-0.403	0.281	-0.351	0.726
	Carling et al. (1995)2	0.128	0.175	0.030	-0.215	0.470	0.730	0.465
	Carling et al. (1995)3	-0.101	0.174	0.030	-0.443	0.241	-0.579	0.563
	Carling et al. (1995)4	0.029	0.174	0.030	-0.312	0.371	0.168	0.867
	Cerqueira et al. (2015)1	-0.782	0.333	0.111	-1.434	-0.129	-2.347	0.019
	Cerqueira et al. (2015)2	-0.556	0.321	0.103	-1.186	0.074	-1.731	0.084
	Cerqueira et al. (2015)3	-0.555	0.321	0.103	-1.185	0.074	-1.728	0.084
	Cerqueira et al. (2015)4	-0.908	0.341	0.116	-1.577	-0.240	-2.662	0.008
	Ferguson et al. (2014)A1	0.032	0.095	0.009	-0.155	0.218	0.332	0.740
	Ferguson et al. (2014)A2	-0.103	0.095	0.009	-0.290	0.083	-1.084	0.278
	Ferguson et al. (2014)A3	-0.022	0.095	0.009	-0.209	0.164	-0.234	0.815
	Ferguson et al. (2014)A4	0.005	0.095	0.009	-0.182	0.191	0.048	0.962
	Ferguson et al. (2014)A5	0.140	0.095	0.009	-0.048	0.327	1.462	0.144
	Ferguson et al. (2014)B1	0.060	0.095	0.009	-0.126	0.247	0.634	0.526
	Ferguson et al. (2014)B2	0.016	0.095	0.009	-0.170	0.203	0.172	0.863
	Ferguson et al. (2014)B3	-0.277	0.096	0.009	-0.465	-0.088	-2.876	0.004
	Ferguson et al. (2014)B4	-0.085	0.095	0.009	-0.272	0.102	-0.892	0.372
	Ferguson et al. (2014)B5	0.066	0.095	0.009	-0.120	0.253	0.697	0.486
	Duffield et al. (2010)A1	0.116	0.182	0.033	-0.241	0.473	0.639	0.523
	Duffield et al. (2010)A2	-0.067	0.182	0.033	-0.424	0.289	-0.371	0.711
	Duffield et al. (2010)A3	-0.155	0.182	0.033	-0.513	0.202	-0.851	0.395
	Duffield et al. (2010)B1	-0.149	0.182	0.033	-0.507	0.208	-0.819	0.413
	Duffield et al. (2010)B2	-0.301	0.184	0.034	-0.661	0.060	-1.636	0.102
	Duffield et al. (2010)B3	-0.170	0.182	0.033	-0.527	0.188	-0.930	0.352
	Jakeman et al. (2010)1	0.030	0.184	0.034	-0.330	0.391	0.164	0.870
	Jakeman et al. (2010)2	-0.470	0.188	0.035	-0.839	-0.101	-2.499	0.012
	Jakeman et al. (2010)3	-0.901	0.200	0.040	-1.293	-0.509	-4.503	0.000
	Jakeman et al. (2010)4	-0.633	0.192	0.037	-1.009	-0.257	-3.299	0.001
	Jakeman et al. (2010)5	-0.593	0.191	0.036	-0.967	-0.218	-3.105	0.002
	Sambaher et al. (2016)	0.015	0.133	0.018	-0.247	0.276	0.110	0.912
	Négyesi et al. (2020)1	-0.409	0.255	0.065	-0.909	0.091	-1.602	0.109
	Négyesi et al. (2020)2	-0.284	0.253	0.064	-0.779	0.211	-1.126	0.260
	Négyesi et al. (2020)3	-1.017	0.282	0.080	-1.570	-0.464	-3.603	0.000
	Ravier et al. (2018)1	-1.235	0.132	0.018	-1.495	-0.976	-9.338	0.000
	Ravier et al. (2018)2	-0.608	0.116	0.014	-0.836	-0.381	-5.233	0.000
	Bieuzen et al. (2014)A1	-0.686	0.193	0.037	-1.063	-0.308	-3.561	0.000
	Bieuzen et al. (2014)A2	-0.587	0.190	0.036	-0.958	-0.215	-3.093	0.002
	Bieuzen et al. (2014)A3	-0.271	0.183	0.034	-0.631	0.089	-1.477	0.140
	Bieuzen et al. (2014)B1	-0.146	0.182	0.033	-0.503	0.211	-0.800	0.423
	Bieuzen et al. (2014)B2	-0.826	0.197	0.039	-1.213	-0.439	-4.185	0.000
	Bieuzen et al. (2014)B3	0.210	0.183	0.033	-0.149	0.568	1.147	0.251
	Chan et al. (2016)A1	0.535	0.207	0.043	0.129	0.941	2.581	0.010
	Chan et al. (2016)A2	0.381	0.204	0.042	-0.019	0.781	1.868	0.062
	Chan et al. (2016)B1	0.387	0.204	0.042	-0.013	0.786	1.898	0.058
	Chan et al. (2016)B2	0.292	0.202	0.041	-0.104	0.688	1.445	0.148
	Kato et al. (2017)	-0.120	0.125	0.016	-0.365	0.125	-0.959	0.338
	Vercruyssen et al. (2017)	-0.221	0.168	0.028	-0.549	0.108	-1.315	0.188
	Xue et al. (2023)1	-1.955	0.369	0.136	-2.679	-1.231	-5.292	0.000
	Xue et al. (2023)2	-2.243	0.407	0.166	-3.041	-1.445	-5.508	0.000
	Xue et al. (2023)3	-1.633	0.333	0.111	-2.286	-0.980	-4.899	0.000
	Xue et al. (2023)4	-1.656	0.336	0.113	-2.314	-0.998	-4.934	0.000
Fixed		-0.182	0.020	0.000	-0.220	-0.143	-9.310	0.000
Random		-0.278	0.049	0.002	-0.375	-0.181	-5.633	0.000

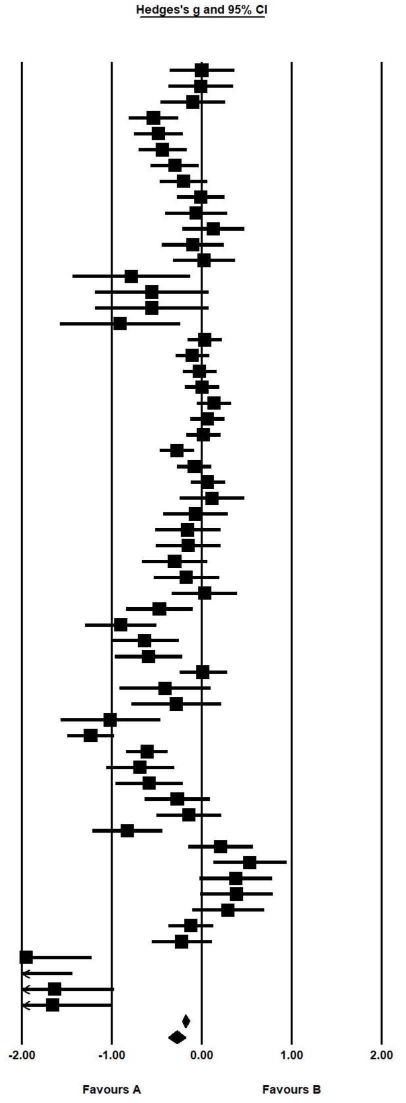

Favours A: Favours [experimental]; Favours B: Favours [control].

Figure S2 Meta-analysis results of the effects of effects of compression garments on power after exercise-induced muscle fatigue

Study name	Hedges's g	Standard error	Variance	Lower limit	Upper limit	Z-Value	p-Value
Atkins et al. (2020)1	0.057	0.133	0.018	-0.204	0.319	0.429	0.668
Atkins et al. (2020)2	0.019	0.133	0.018	-0.242	0.280	0.142	0.887
Davies et al. (2009)	-0.037	0.182	0.033	-0.393	0.319	-0.203	0.839
Govus et al. (2018)1	0.063	0.191	0.036	-0.312	0.437	0.328	0.743
Govus et al. (2018)2	0.213	0.192	0.037	-0.163	0.589	1.110	0.267
Govus et al. (2018)3	0.114	0.191	0.037	-0.260	0.489	0.598	0.550
Govus et al. (2018)4	-0.021	0.191	0.036	-0.395	0.353	-0.109	0.913
Zinner et al. (2017)A	0.332	0.169	0.029	0.001	0.663	1.966	0.049
Zinner et al. (2017)B	0.085	0.167	0.028	-0.242	0.412	0.510	0.610
O¡¯Riordan et al. (2022)1	0.000	0.286	0.082	-0.560	0.560	0.000	1.000
O¡¯Riordan et al. (2022)2	-0.395	0.291	0.085	-0.966	0.176	-1.355	0.175
O¡¯Riordan et al. (2022)3	-0.197	0.287	0.082	-0.760	0.365	-0.687	0.492
O¡¯Riordan et al. (2022)4	-0.197	0.287	0.082	-0.760	0.365	-0.687	0.492
Jakeman et al. (2010)A1	-0.049	0.184	0.034	-0.409	0.311	-0.266	0.790
Jakeman et al. (2010)A2	-0.418	0.187	0.035	-0.785	-0.051	-2.233	0.026
Jakeman et al. (2010)A3	-0.588	0.191	0.036	-0.962	-0.214	-3.082	0.002
Jakeman et al. (2010)A4	-0.424	0.187	0.035	-0.791	-0.057	-2.262	0.024
Jakeman et al. (2010)A5	0.043	0.184	0.034	-0.317	0.404	0.235	0.814
Jakeman et al. (2010)B1	-0.448	0.188	0.035	-0.817	-0.080	-2.387	0.017
Jakeman et al. (2010)B2	-0.963	0.202	0.041	-1.360	-0.567	-4.760	0.000
Jakeman et al. (2010)B3	-0.984	0.203	0.041	-1.383	-0.586	-4.845	0.000
Jakeman et al. (2010)B4	-0.706	0.194	0.038	-1.086	-0.326	-3.642	0.000
Jakeman et al. (2010)B5	-0.475	0.188	0.035	-0.844	-0.106	-2.523	0.012
Bieuzen et al. (2014)A1	-0.182	0.183	0.033	-0.539	0.176	-0.995	0.320
Bieuzen et al. (2014)A2	-0.610	0.190	0.036	-0.983	-0.237	-3.207	0.001
Bieuzen et al. (2014)A3	-1.179	0.213	0.046	-1.597	-0.760	-5.523	0.000
Bieuzen et al. (2014)B1	-0.572	0.189	0.036	-0.943	-0.201	-3.022	0.003
Bieuzen et al. (2014)B2	-0.499	0.187	0.035	-0.867	-0.132	-2.662	0.008
Bieuzen et al. (2014)B3	-0.876	0.199	0.040	-1.266	-0.485	-4.395	0.000
Ehrstrom et al. (2018)1	-0.322	0.156	0.024	-0.628	-0.017	-2.067	0.039
Ali et al. (2011)A	-0.429	0.171	0.029	-0.763	-0.095	-2.517	0.012
Ali et al. (2011)B	-0.531	0.173	0.030	-0.869	-0.193	-3.079	0.002
Ali et al. (2011)C	-0.260	0.168	0.028	-0.590	0.069	-1.549	0.121
Bindemann (2007)	0.000	0.286	0.082	-0.560	0.560	0.000	1.000
Areces et al. (2015)	0.434	0.120	0.015	0.198	0.670	3.604	0.000
Coso et al. (2014)	0.232	0.112	0.013	0.012	0.451	2.064	0.039
Ali et al. (2010)A1	-0.038	0.200	0.040	-0.430	0.354	-0.192	0.848
Ali et al. (2010)A2	-0.009	0.200	0.040	-0.401	0.383	-0.047	0.962
Ali et al. (2010)A3	0.000	0.200	0.040	-0.392	0.392	0.000	1.000
Ali et al. (2010)B1	-0.101	0.200	0.040	-0.494	0.291	-0.506	0.613
Ali et al. (2010)B2	-0.081	0.200	0.040	-0.473	0.311	-0.404	0.686
Ali et al. (2010)B3	-0.060	0.200	0.040	-0.452	0.332	-0.300	0.764
Rugg et al. (2013)	-0.234	0.144	0.021	-0.516	0.048	-1.625	0.104
Shen et al. (2021)A	-0.934	0.222	0.049	-1.369	-0.500	-4.212	0.000
Shen et al. (2021)B	-0.903	0.220	0.049	-1.335	-0.471	-4.096	0.000
Shen et al. (2021)C	0.025	0.200	0.040	-0.367	0.417	0.127	0.899
Shen et al. (2021)D	1.263	0.240	0.058	0.793	1.733	5.265	0.000
Ehrstrom et al. (2018)2	-0.219	0.155	0.024	-0.523	0.085	-1.413	0.158
	-0.227	0.060	0.004	-0.344	-0.109	-3.775	0.000

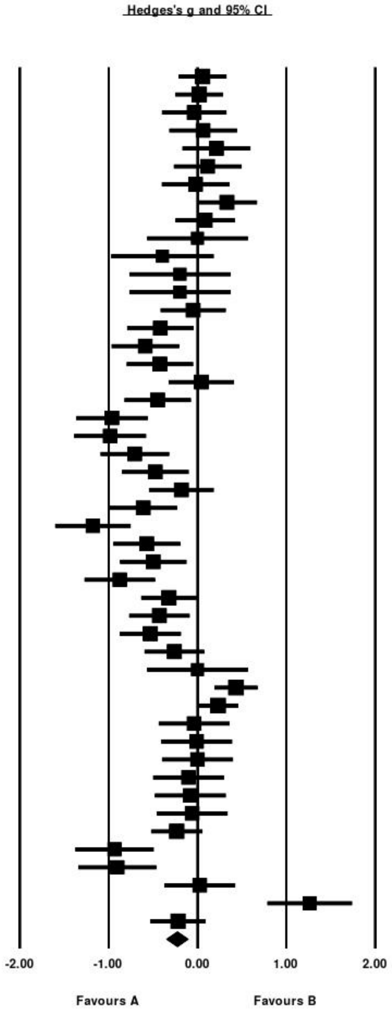

Favours A: Favours [experimental]; Favours B: Favours [control].

Figure S3 Funnel plots

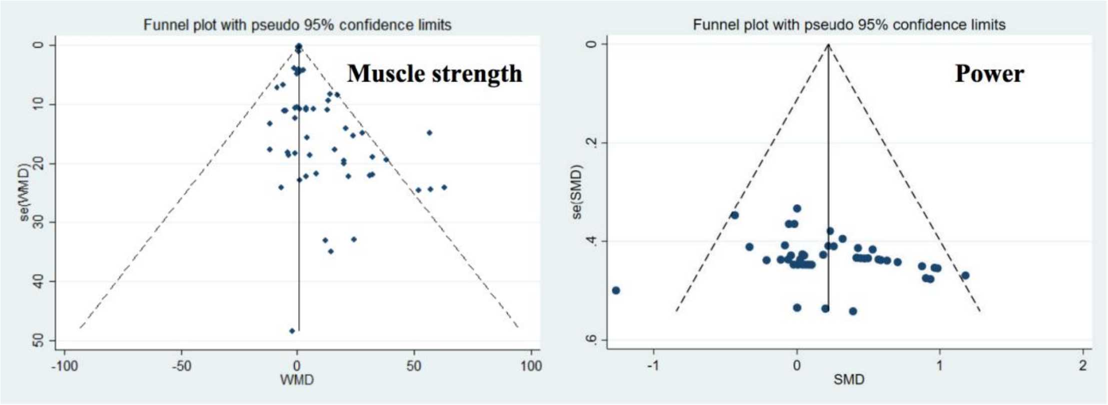

Figure S4 Sensitivity analyses results of muscle strength

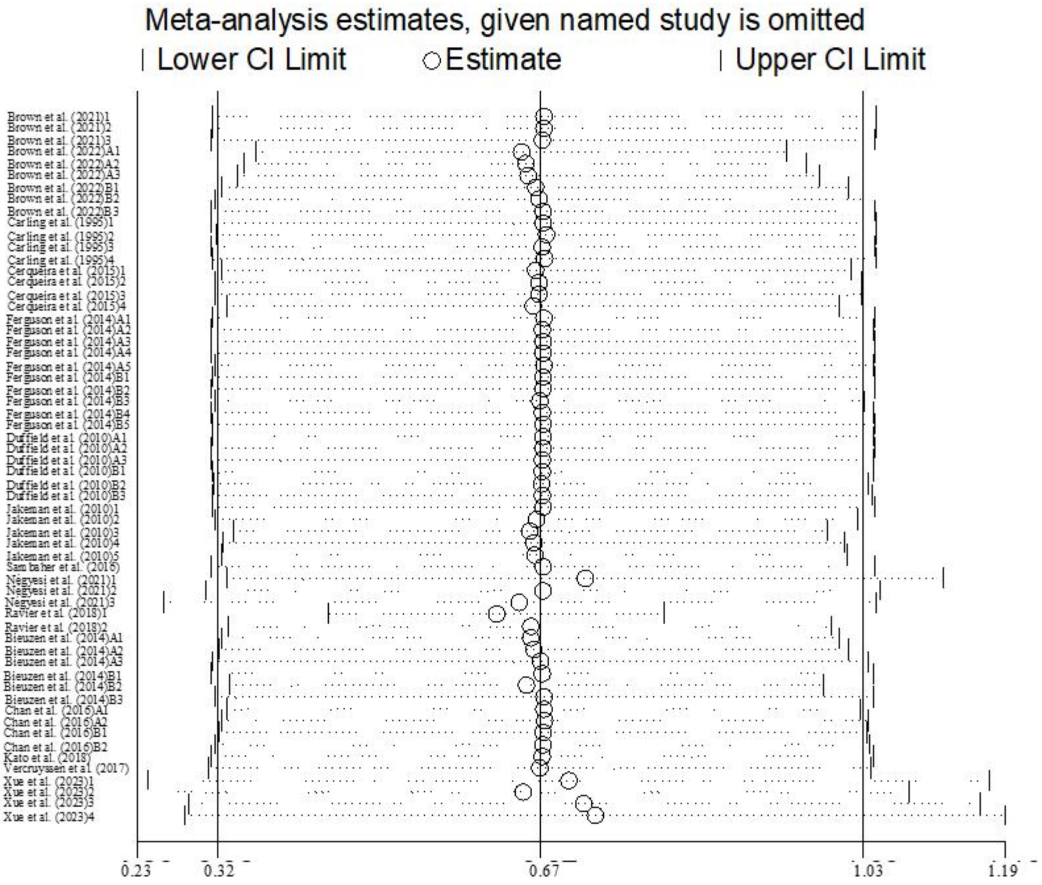

Figure S5 Sensitivity analyses results of power

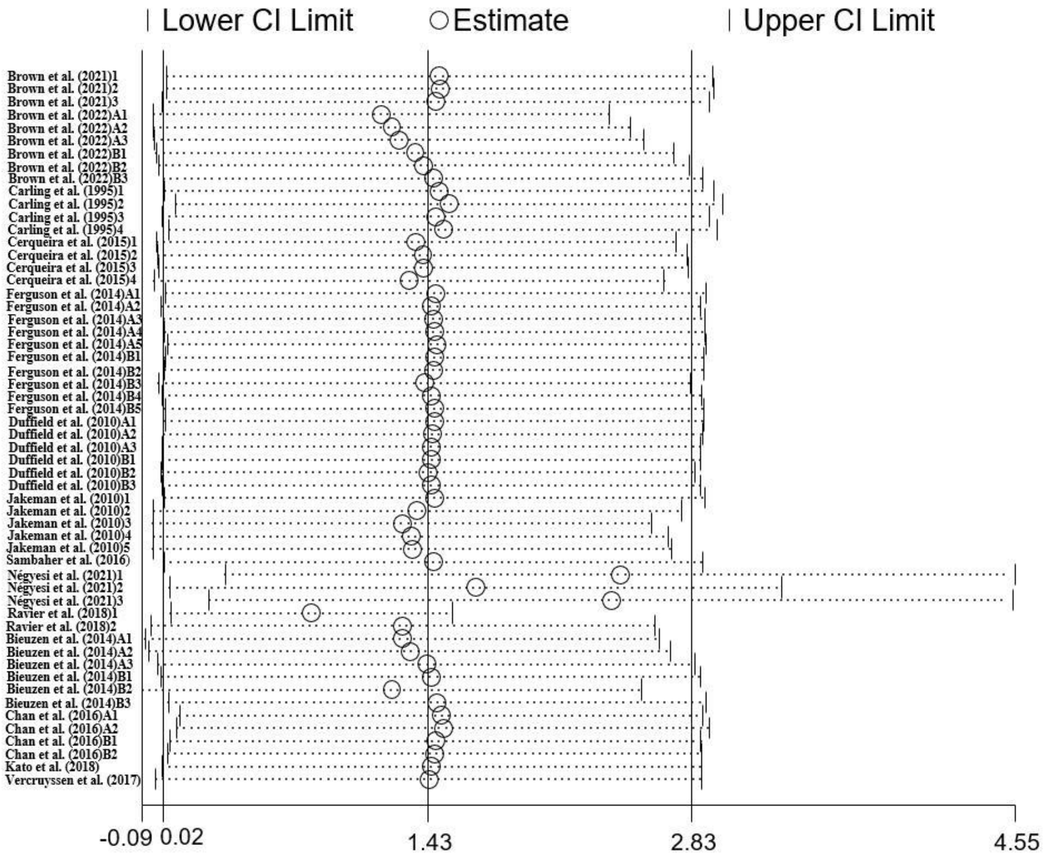

Table S1 Search terms for compression garment, muscle strength, and power

**Compression garment search terms**
compression garments OR compression garment OR compression clothes OR compression clothe OR compression clothing OR compression uniforms OR compression uniform OR compression socks OR compression stockings OR compression sleeves
**Muscle strength search terms**
muscle strength OR strength, muscle OR muscle force OR muscle contraction OR maximal strength OR repetition maximum OR maximal voluntary contraction OR maximal isokinetic torque OR resistance exercise OR resistance training OR strength training OR resistance exercise OR resistance training OR strength training OR torque
**Power search terms**
explosive strength OR power OR muscle power OR torque-velocity OR power output OR force-velocity OR rate of force development OR explosive training OR explosive exercise OR power training OR vertical jump OR countermovement jump OR squat jump OR plyometrics OR height OR drop jump OR depth jump OR jump training

Table S2 Characteristics of the studies included in this meta-analysis

Study	Participants	Sample size	Age (years)	Types of compression garments		Experimental program		Main outcomes
				Typology	Pressure	Fatigue training	Time in compression garments	
Atkins et al. (2020)	Basketball players	Men: 30	22.5 ± 4.1	Full-length, lower-body compression garments	Ankle: 7 ± 3 mmHg; Calf: 10 ± 3 mmHg; Thigh: 8 ± 2 mmHg	2 sets of 12-minute basketball-specific training programs (including vertical jumps, jogging, acceleration runs, deceleration runs. high shuffles, low shuffles), 4 sets of 10 lunge jumps, 1 set of 2-minute static squats against the wall	Wearing full-length, lower-body compression garments for 15 h recovery after exercise	CMJ (immediate and 15 h post-exercise)
Brown et al. (2021)	Judo players	Men: 5 Women: 6	Men: 21 ± 2 Women: 23 ± 2	Custom-fitted full length compression stockings and arm compression sleeves	NR	28 min of randori per judoka	Wearing custom-fitted full length compression stockings and arm compression	MVC (12 h, 36 h, and 43 h post-exercise)

							sleeves for 24 h recovery after exercise	
Brown et al. (2022)	Football players	Men: 45	23 ± 4	Customized lower limb compression garments or suitably sized lower limb compression garments	NR	20 sets of 20m sprint run, 5m deceleration run, 100 drop jumps	Wearing customized lower limb compression garments or suitably sized lower limb compression garments for 48 h recovery after exercise	MVC (immediate, 24 h, and 48 h post-exercise)
Carling et al. (1995)	No training experience university students	Men: 7 Women: 16	26 ± 4	Compression sleeves	17 mmHg	70 centrifugal contractions of non-dominant elbow flexor	Wearing compression sleeves for 72 h recovery after exercise	MVC (10 min, 24 h, 48 h, and 72 h post-exercise)
Cerqueira et al. (2015)	No training experience people	Men: 13	21 ± 1	Compression sleeves	NR	30 elbow curls centrifugal contraction	Wearing compression sleeves for 12 h recovery after exercise	MVC (24 h, 48 h, 72 h, and 96 h post-exercise)
Davies et al. (2009)	Basketball players	Men: 4 Women:	22.1 ± 4.4	Compression tights	NR	5 sets of 20 reps of 60cm height drop	Wearing compression	CMJ (48 h post-exercise)

		7				jump	tights for 48 h recovery after exercise	
Ferguson et al. (2014)	Athletes in Football and rugby	Men: 21	21 ± 1	Graduated compression socks	Ankle: 40 mmHg Calf: 20 mmHg	2 sets 45-min sections of continuous intermittent exercise, 3 × 20 m at walking pace, 1 × 20 m maximal sprint, 4 s recovery	Wearing graduated compression socks for 12 h recovery after exercise	MVC (immediate, 1 h, 24 h, 48 h, and 72 h post-exercise)
Govus et al. (2018)	Cross-country skiers	Men: 18 Women: 14	22.2 ± 4.0	Compression tights	Ankle: 14.6 ± 0.1 mmHg; Achilles tendon: 13.6 ± 1.4 mmHg; Calf: 13.7 ± 1.3 mmHg; Tibial tuberosity: 7.6 ± 1.1 mmHg;	4 sets 1570 m cross-country sprint skiing competition	Recovering in compression tights after a cross-country ski race for about 17 h	CMJ (8 h, 20 h, 44 h, 68 h post-exercise)

					Patella: 8.3 ± 2.3 mmHg; Mid-thigh: 5.3 ± 1.1 mmHg; 5 cm below crotch: 4.2 ± 2.8 mmHg			
Zinner et al. (2017)	Handball players	Total: 20	22 ± 4	Compression pants	0 mmHg COMP group: 0 mmHg; 10 mmHg COMP group: 10 mmHg; 25 mmHg COMP group: 25 mmHg	3 sets of 30 reps 30 m sprints and 2 countermovement jumps	Wearing different type compression pants for 48 h recovery after exercise	CMJ (48h post-exercise)
O'Riordan et al. (2022)	Resistance-trained participants	Men: 13 Women: 9	25.9 ± 5.0	Compression tights	NR	8 sets of 6 reps of 85%1RM eccentric focused leg press exercise	Wearing compression tights for 4 h recovery after exercise	CMJ (immediate, 4 h, 24 h, 48 h post-exercise)
Duffield et al. (2010)	Team-sport athletes	Total: 11	20.9 ± 2.7	Compression tights	NR	10 sets of 20 m sprint run, 10m deceleration runs and 10 double leg bounds	Wearing compression tights during exercise and wear compression	MVC (immediate, 2 h, 24 h post-exercise)

							tights after exercise for 24 h recovery after exercise	
Jakeman et al. (2010)	Female volunteers	Women: 32	21.4 ± 1.7	Compression tights	Calf: 17.3 mmHg Tight: 14.9 mmHg	110 sets of 10 reps of 60 cm drop jumps	Wearing compression tights for 12 h recovery after exercise	CMJ, SJ, and MVC (1 h, 24 h, 48 h, 72 h, 96 h post-exercise)
Šambaher et al. (2016)	University students	Men: 7 Women: 8	23.5 ± 3.2	Ankle compression sleeves	Ankle: 20 mmHg Knee: 30 mmHg	Continuous drop jumps from a 30-cm platform at 70 Hz in accordance with a metronome	Wearing ankle compression sleeves during exercise	MVC (immediate post-exercise)
N é gyesi et al. (2021)	Healthy adults	Men: 12 Women: 12	25.5 ± 4	Below-knee compression garments	NR	100 maximal isokinetic eccentric contractions with the extensors of the right-dominant leg at 30° /s.	Wearing below-knee compression garments during exercise	MVC (immediate, 24 h, and 1week post-exercise)
Ravier et al. (2018)	Handball players	Men: 18	23.22 ± 4.97	Full-leg Compression garments	Ankle: 15 mmHg Calf: 27 mmHg Tight: 14 mmHg	33 sets of handball-specific circuits (W-sprints, lateral-side cone	Wearing full-leg compression garments	MVC (immediate and 24 h post-exercise)

						drill, 15 m sprint, 8 plyometric single-leg jumps and throws, 4 CMJ)	during exercise	
Bieuzen et al. (2014)	Highly trained male runners	Men: 11	34.7 ± 9.8	Compression calf stockings	20 mmHg compression calf socks: 20 mmHg; 15 mmHg compression calf socks: 15 mmHg	15.6 km simulated trail race	Wearing tight calf socks during exercise and wear tight calf socks after exercise for 24 h	CMJ and MVC (immediate, 24 h, and 48 h post-exercise)
Chan et al. (2016)	Healthy male participants	Men: 10	23 ± 3	Long-sleeve compression top and short-leg compression pants	NR	2 sets of 10 movements in a circuit exercise (50 stairs total), log weight carry, log weight carry, tyre stack, sledgehammering, vibration platform, box carrying, overhead lifting, trolley, heavy rope pull, each movement lasts 9	Wearing long-sleeve compression top and short-leg compression pants during exercise	MVC (immediate and 24 h post-exercise)

						minutes		
Kato et al. (2018)	Healthy adults	Men: 9 Women: 7	33.3 ± 5.4	Lower limb compression garments	NR	20 heel-rise exercises with right lower limb with the knee extended on a step wedge (height 20 cm)	Wearing lower limb compression garments during exercise	MVC (immediate post-exercise)
Vercruyssen et al. (2017)	Competitive trail runners	Total: 12	39.6 ± 4.6	Compression stocking	Ankle: 18 mmHg; Calf: 13 mmHg	2 sets 18.4-km short distance trail runs	Wearing compression stocking during exercise	MVC (immediate post-exercise)
Xue et al. (2023)	Healthy male participants	Total: 16	19.75 ± 1.75	Lower limb compression sleeves	NR	Participants will complete 5 sets of 20 continuous drop jumps from a 0.6 m box, with 10-second intervals and 2-minute rest periods between sets.	Wearing graduated Lower limb compression sleeves during exercise	MVC (immediate, 24 h, and 48 h post-exercise)
Ehrstrom et al. (2018)	Well-trained male trail runners	Men: 13	38.6 ± 5.7	Lower limb compression garments	Middle of calf: 20-25mmHg; Upper site of calf: 18-20mmHg;	3 minutes of high-intensity level running (i.e. corresponding to	Wearing lower limb compression garments	CMJ (immediate post-exercise)

					Lower site of thigh:18-20mmHg	3.88 m/s). Treadmill slope was immediately set to a -8.5° and VDHR was also set to induce the equivalent of a metabolic intensity of 55% VO_2max	during exercise	
Ali et al. (2011)	Well-trained, competitive runners	Total: 11	33 ± 10	Graduated compression stockings	Low grade GCS group: ankle: 15 mmHg, knee: 12 mmHg; Medium grade GCS group: ankle: 21 mmHg, knee: 18 mmHg; High grade GCS group: ankle: 32 mmHg, knee: 23 mmHg	10-km running	Wearing graduated compression stockings during exercise	CMJ (immediate post-exercise)
Bindemann (2007)	Running players	Men: 7	43.7 ± 5.5	Compression stockings	Ankle: 32 mmHg Below the knee: 23 mmHg	90-minute variant gradient run, followed by a 30-minute	Wearing compression stockings during exercise	CMJ (immediate post-exercise)

						downhill run		
Areces et al. (2015)	Marathon runners	Men: 30 Women: 4	41.95 ± 8.28	Compression stockings	20 mmHg -25 mmHg	Finish a marathon race	Wearing compression stockings during the race	CMJ (immediate post-exercise)
Coso et al. (2014)	Experienced triathletes	Total: 36	35.38 ± 5.72	Compression stockings	NR	Finish a half-ironman triathlon race	Wearing compression stockings during exercise	CMJ (immediate post-exercise)
Shen et al. (2021)	Healthy women who often exercise	Women: 10	22.8 ± 1.4	Compression socks	4 types of compression socks: 10mmHg; 15mmHg; 20mmHg; 25mmHg	Walked for 3 min at the speed of 2.7 km/h on 0 gradient. then gradually increase the load until fatigued, and unable to walk any longer	Wearing compression socks during exercise	CMJ (immediate post-exercise)
Ali et al. (2010)	People who exercise regularly	Men: 9 Women: 1	36 ± 10	Graduated compression stockings	Low graduated compression stockings: ankle: 15 mmHg, knee: 12 mmHg; High graduated compression stockings: ankle: 32	40-min treadmill running	Wearing graduated compression stockings during exercise	CMJ (immediate, 24 h, and 48 h post-exercise)

					mmHg, knee: 23 mmHg			
Rugg et al. (2013)	Collegiate-level track and field athletes	Men: 8 Women: 6	28.2 ± 14.0	Graduated compression tights	Ankle: 18mmHg Calf: 12.6mmHg Thigh: 7.2mmHg	Each trial consisted of 15 minutes of continuous running with 5 minutes performed at each of the following intensities: 50%, 70%, and 85% HRR	Wearing graduated compression stockings during exercise	CMJ (immediate post-exercise)

Abbreviations: MVC, muscle maximal voluntary contraction; CMJ, countermovement jump; SJ, Squat jump; 1RM, one-repetition maximum; reps, repetitions; HRR, heart rate reserve; NR, no report.

Table S3 Results of meta-regression

Measurements	_ES	Coef.	Std. Err	t	*P* > |t|	95% CI
Strength	**Rest intervals**					
	subgroup	0.0422968	0.0485487	0.87	0.387	-0.0546339, 0.1392275
	_cons	0.1460554	0.1222387	1.19	0.236	-0.098002, 0.3901127
	**Body parts**					
	subgroup	0.0645113	0.1589049	0.41	0.686	-0.2527525, 0.3817751
	_cons	0.1214131	0.3033809	0.40	0.690	-0.4843065, 0.7271327
	**Training experience**					
	subgroup	0.1476915	0.146819	1.01	0.318	-0.145442, 0.440825
	_cons	0.0743815	0.1750211	0.42	0.672	-0.2750595, -0.2750595
	**Rest intervals at the upper limb**					
	subgroup	0.1554899	0.1591902	0.98	0.354	-0.2046233, 0.5156032
	_cons	-0.2641465	0.4814603	-0.55	0.597	-1.353285, 0.8249923
	**Rest intervals at the lower limb**					
	subgroup	0.0386056	0.0548212	0.70	0.484	-0.0712585, 0.1484696
	_cons	0.1652773	0.133867	1.23	0.222	-0.1029983, 0.4335528
	**Body parts at trained individuals**					
	subgroup	0.1935367	0.2796273	0.69	0.492	-0.3673246, 0.7543981
	_cons	-0.1564405	0.5499007	-0.28	0.777	-1.259402, 0.9465206
	**Body parts at untrained individuals**					
	subgroup	0.2649486	0.2590293	1.02	0.328	-0.305171, 0.8350681
	_cons	-0.008555	0.3914423	-0.02	0.983	-0.8701138, 0.8530038
Power	**Rest intervals**					
	subgroup	0.067156	0.05835	1.15	0.256	-0.0503668, 0.1846789
	_cons	0.0752995	0.1391312	0.54	0.591	-0.2049252, 0.3555242

Abbreviations: Coef., coefficient; Std. Err., standard error; t, t-test statistic; CI, confidence interval.

Table S4 Results of Egger's test

Measurements	Std_EFF	Coef.	Std. Err.	t	P > |t|	95% CI
**Strength**	Slope	0.476997	0.0995985	4.79	< 0.001	0.277629, 0.676365
	Bias	0.5510895	0.1358299	4.06	< 0.001	0.2791965, 0.8229825
**Power**	Slope	-0.6674752	0.5733102	-1.16	0.250	-1.821489, 0.4865383
	Bias	2.055389	1.322307	1.55	0.127	-0.606277, 4.717055

Abbreviations: Coef., coefficient; Std. Err., standard error; t, t-test statistic; CI, confidence interval.

Table S5 Results of trim and fill analysis

Method	Pooled Est	95% CI		Asymptotic		No. of studies
**Meta-analysis**						
		Lower	Upper	z-value	P-value	
Fixed	0.627	0.441	0.812	6.619	< 0.001	60
Random	0.673	0.316	1.031	3.691	< 0.001	
**Filled meta-analysis**						
Method	Pooled Est	95% CI		Asymptotic		No. of studies
		Lower	Upper	z-value	P-value	
Fixed	0.615	0.429	0.800	6.493	< 0.001	75
Random	0.646	0.045	1.247	2.106	0.035	

Abbreviations: CI, confidence interval.
